# Elucidating the causal links between plasma and cerebrospinal fluid metabolites and pituitary tumors: a Mendelian randomization analysis

**DOI:** 10.3389/fendo.2024.1460278

**Published:** 2024-11-28

**Authors:** Wencai Wang, Menghao Liu, Zun Wang, Wei Ye, Xianfeng Li

**Affiliations:** Department of Neurosurgery, The Second Affiliated Hospital of Harbin Medical University, Harbin, China

**Keywords:** Mendelian randomization, 3-dehydrocarnitine, acetylcarnitine, pituitary tumors, metabolites, pathogenesis

## Abstract

**Background:**

Pituitary tumors (PTs) are common benign intracranial tumors. Investigating the metabolites in serum and cerebrospinal fluid in PTs is essential to understanding the underlying biological mechanisms and identifying new biomarkers and therapeutic strategies.

**Methods:**

We used the GWAS dataset of PTs from the FinnGen database, a dataset of 486 plasma metabolites from the GWAS catalog database, and a dataset of 338 cerebrospinal fluid (CSF) metabolites from the WADRC and WRAP study collections. An inverse variance weighting (IVW) approach was utilized as the mainly method to investigate causality between metabolites and PTs, supplemented by four complementary methods to strengthen our findings. Additionally, we utilized several sensitivity methods to guarantee the robustness of our findings.

**Results:**

The study identified 17 plasma metabolites and 10 CSF metabolites related to PTs. Among these, 11 metabolites indicated a significant positive causality with PTs, while 16 displayed a remarkable negative causality. Particularly, plasma levels of 3-dehydrocarnitine (OR = 2.73, 95% CI = 1.55–4.83, P = 0.001) and acetylcarnitine (OR = 0.35, 95% CI = 0.19–0.63, P = 0.001) were found to be significant exposure factors for PTs. Multiple sensitivity analyses confirm the robustness of the results. The study found no evidence of a reverse causality between PTs and the plasma levels of 3-dehydrocarnitine and acetylcarnitine.

**Conclusions:**

The present study identified 27 metabolites associated with the incidence of PTs, among which 3-dehydrocarnitine and acetylcarnitine are the most noteworthy.

## 1 Introduction

Pituitary tumors (PTs) are the second most common primary brain neoplasm, accounting for about 10-15% of all brain tumors, and are the most common benign tumor in the saddle region (1). Larger PTs often cause patients to experience visual field defects, headaches or hypopituitarism (2). Additionally, functional PTs can produce excessive amounts of hormones, resulting in various endocrine symptoms (3). The first-line treatment option for PTs is typically endoscopic transsphenoidal pituitary surgery, except for prolactinomas, for which the first-line treatment option is medication with bromocriptine or carbamazepine (4).

In recent years, the rapid development of metabolomics has provided new research methods for studying the pathogenesis of diseases, including PTs, and for searching for biomarkers (5). Previous studies have shown that choline is negatively correlated with growth inhibitory receptor type 2 expression in growth hormone-secreting pituitary adenomas, and positively related to magnetic resonance imaging T2 signals and Ki-67 indices (6). In addition, growth inhibitor stimulates BKCa channels in rat PT cells via lipoxygenase metabolites of arachidonic acid (7). Various studies suggest that metabolites are closely related to the development of PTs (8, 9). Nevertheless, it remains unknown which metabolites are causally associated with PTs.

Mendelian randomization (MR) is an epidemiological technique that uses genome-wide association study (GWAS) data to investigate causality between exposures and outcomes (10). It reduces the effects of reverse causality and confounding factors. Therefore, we intended to investigate the effects of serum and cerebrospinal fluid (CSF) metabolites on PTs employing MR.

## 2 Methods

### 2.1 Study design

**Figure 1:**
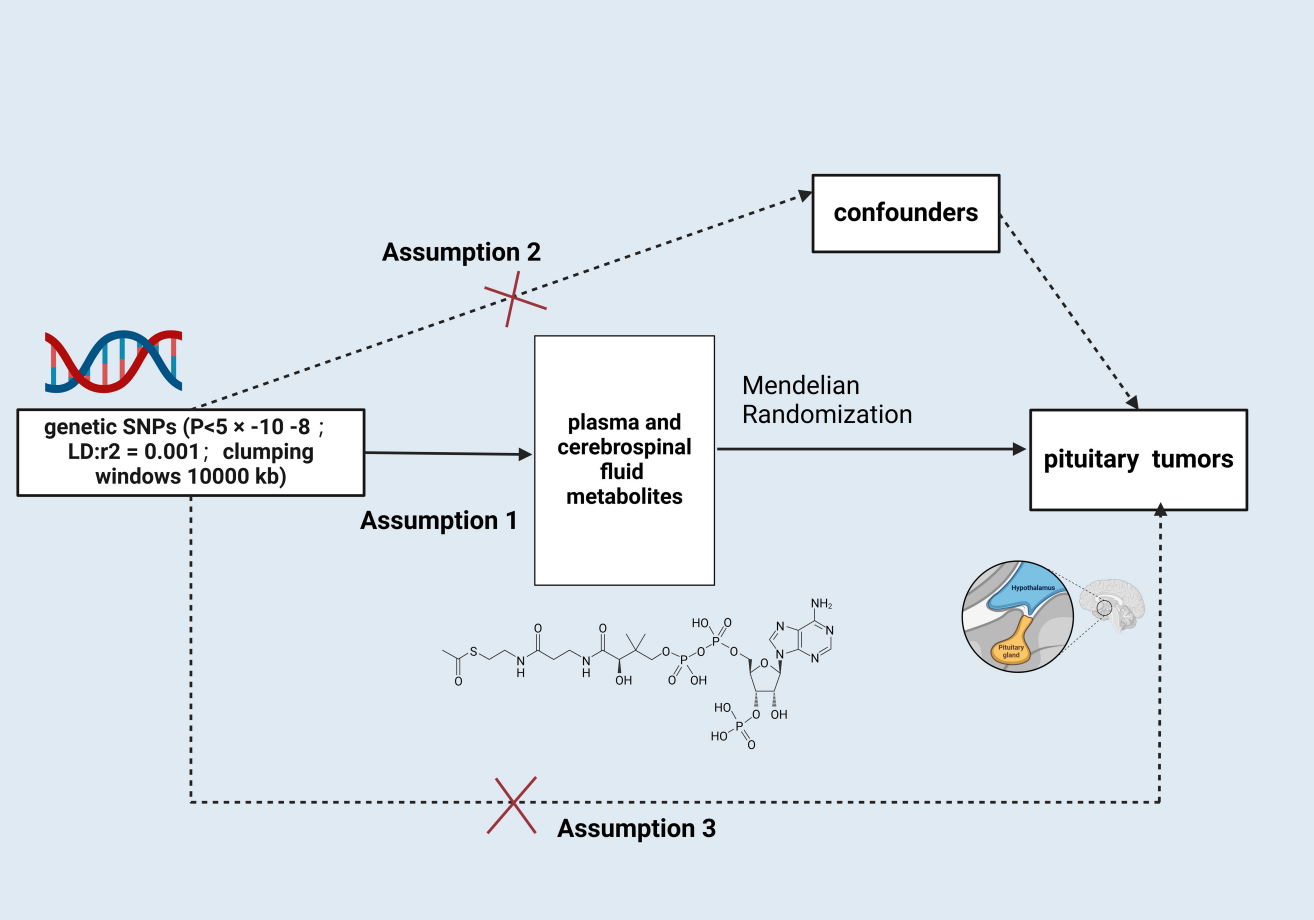
Schematic illustration of the study design. snps, single nucleotide polymorphisms. Created with BioRender.com.

This paper is based on the STROBE-MR report and strictly follows the three main assumptions of MR analysis (11): 1) the designated instrumental variables(IVs) are strongly related to serum and CSF metabolites; 2) the IVs are not confounded by other factors; and 3) the IVs influence the occurrence of PTs only through serum and CSF metabolites, not through other pathways (**Figure 1**).

### 2.2 GWAS data source

The GWAS dataset of PTs as defined by the International Classification of Diseases, 10th Revision (ICD-10) is from round 9 of the FinnGen database. It includes 1,402 European patients with PTs and 375,875 control European patients (12). The serum metabolite GWAS dataset consists primarily of 486 metabolites from the German KORA F4 study (n = 1,768 individuals) and the UK TwinsUK study (n = 6,056 individuals) (13). The 338 CSF Metabolites GWAS dataset was obtained from CSF specimens from 689 participants in the Wisconsin Alzheimer’s Disease Research Centre (532 participants) and the Wisconsin Alzheimer’s Disease Prevention Registry (168 participants). The study received approval from the Institutional Ethics Review Board of the University of Wisconsin Health Sciences (14).

### 2.3 IVs selection

We took the following steps in screening the IVs:1) We used a threshold of p<5×10^−8^ to screen SNPs; 2) We used the criteria of r2<0.001 and LD = 10,000 kb to remove linkage disequilibrium; 3) We calculated the F-statistic for each SNP, excluding those with F-statistics smaller than 10 and retaining those larger than 10; 4) We excluded SNPs in palindromic sequences; 5) Finally, we used PhenoScanner to remove SNPs related to potential confounding factors (15).

### 2.4 Statistical analysis

Our MR analyses were all performed using the TwoSampleMR package in R Studio (16). First, serum and CSF metabolites were selected as IVs, and PTs were considered endpoints. The inverse variance weighting (IVW) technique was used as the mainly analysis method. Furthermore, weighted modal, weighted median, MR-Egger, and simple modal methods were used as supplementary analyses. Given the exploratory nature of this study, we did not apply the Bonferroni correction method; instead, results with a P-value of less than 0.05 were considered significant.

We performed sensitivity analyses of our MR results using a variety of methods.MR-Egger regression intercepts and Mendelian randomized polytomous residuals and outliers (MR-PRESSO) were used to detect potential horizontal pleiotropy. Cochrane’s Q-statistics and their corresponding p-values were computed in the IVW test and MR-Egger regression to evaluate potential heterogeneity among IVs. If significant heterogeneity was found (p<0.05), we used the random effects model of IVW to obtain more unbiased and robust estimates. The robustness of our MR analysis results was verified through scatterplots and funnel plots. Additionally, leave-one-out analyses were conducted to evaluate the potential impact of individual SNPs on the observed causal relationships.

### 2.5 Metabolic pathway analysis

We performed metabolic pathway analyses of metabolites causally associated with PTs using Metaconflict 5.0. (https://www.metaboanalyst.ca/) (17). In summary, 11 metabolic pathways were identified from the metabolite database, with 9 pathways sourced from both the Small Molecule Pathway (SMP) database and the KEGG database, and 2 pathways derived exclusively from KEGG.

## 3 Results

### 3.1 MR analysis of 486 plasma metabolites’ impact on PTs

**Figure 2:**
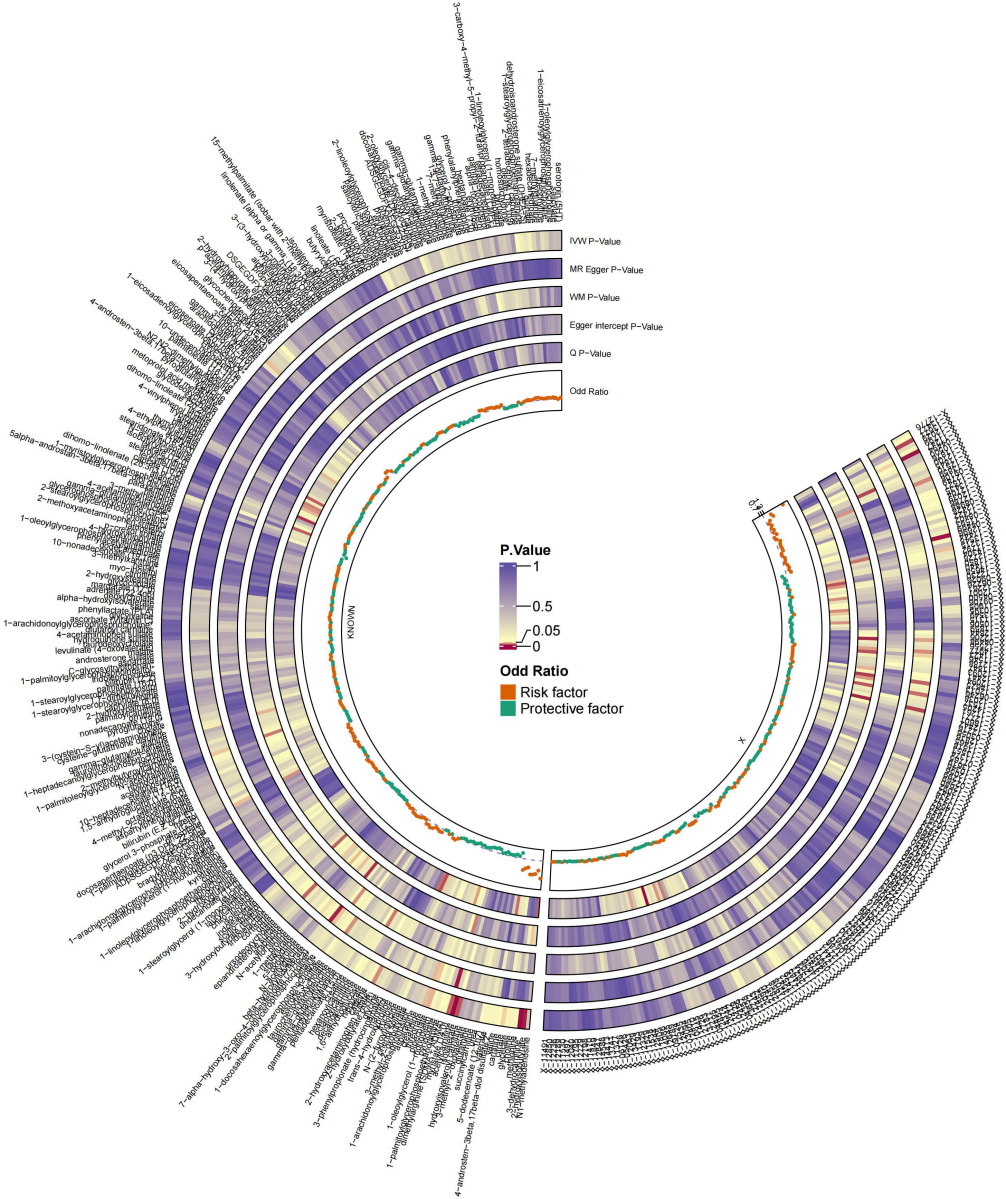
Summary of causal estimates regarding the impact of all plasma metabolites on PTs in MR analysis. From outside to inside, the corresponding P-values of IVW, MR-Egger, WM, Egger intercept, MRPRESSO Global Test, Q, odds ratio are represented, respectively. MR, Mendelian randomization; IVW, inverse-variance weighted; WM, weighted median.

**Figure 3:**
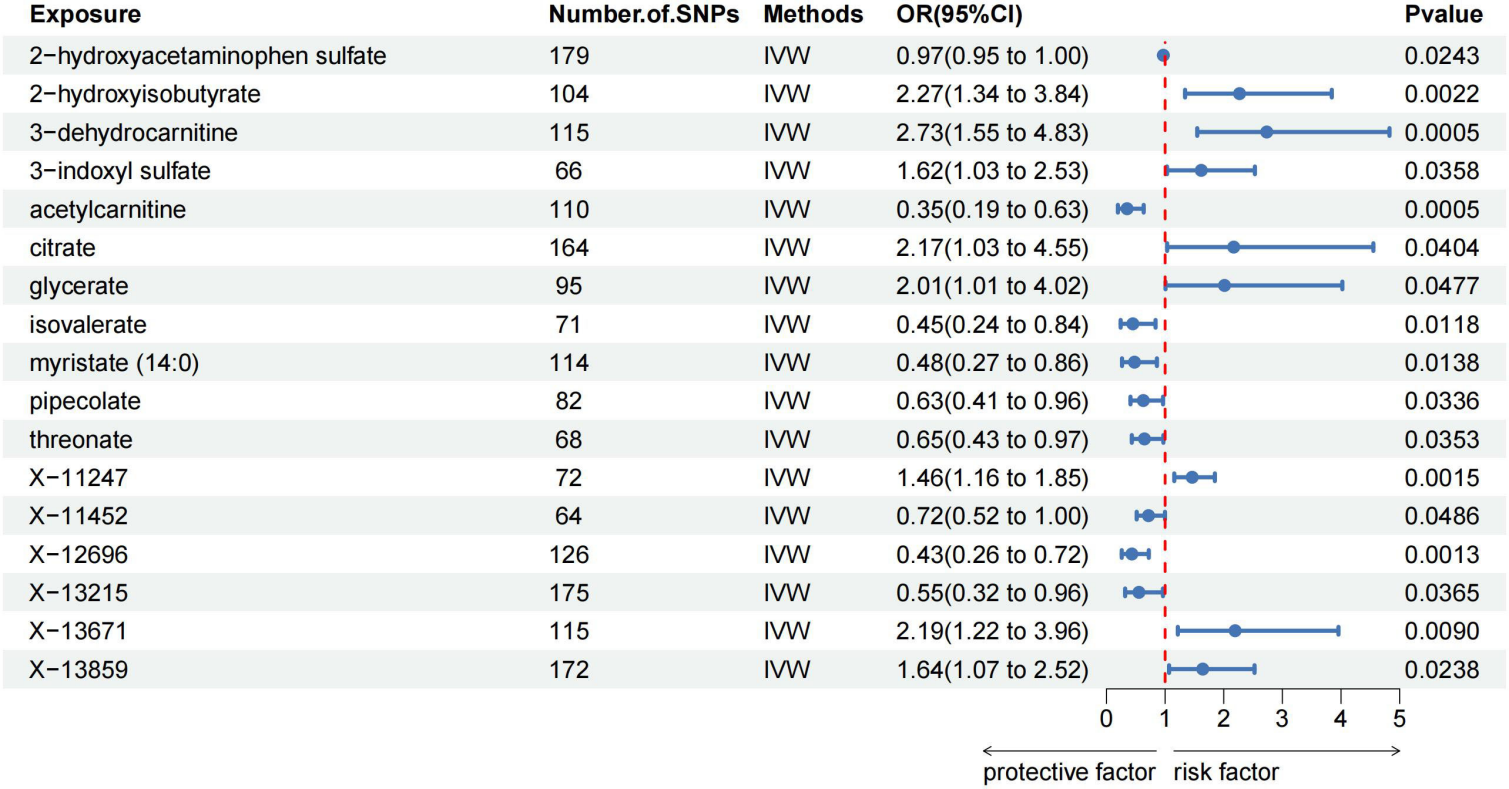
Forest plots illustrating the significant causal estimates of plasma metabolites on PTs are presented. SNPs, single nucleotide polymorphisms; OR, odds ratio; IVW, inverse-variance weighted.

**Figure 4:**
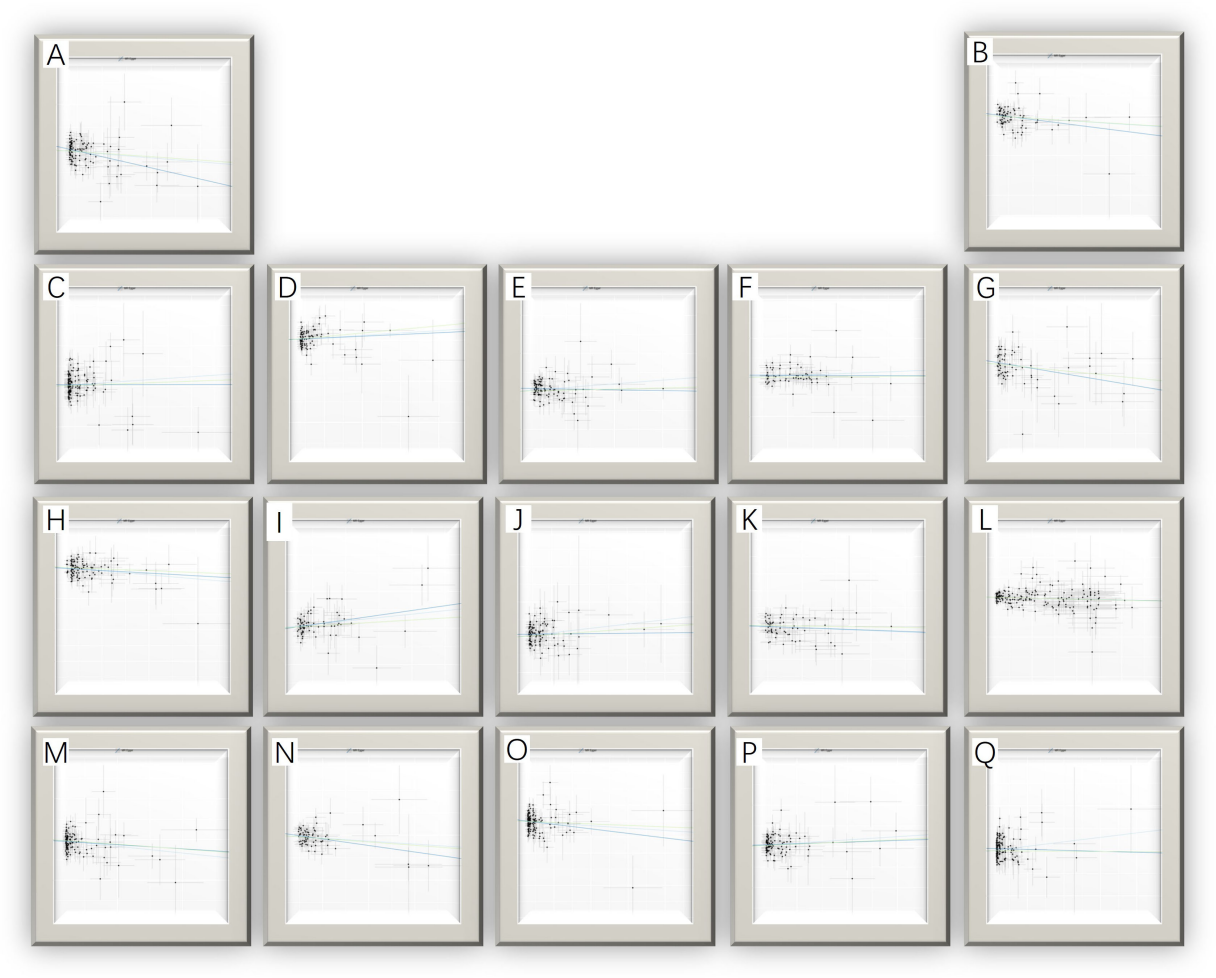
Scatter plots for the causal association between plasma metabolites and PTs. **(A)** myristate (14:0) **(B)** pipecolate **(C)** citrate **(D)** glycerate **(E)** 2-hydroxyisobutyrate **(F)** 3-indoxyl sulfate **(G)** threonate **(H)** acetylcarnitine **(I)** X-11247 **(J)** 3-dehydrocarnitine **(K)** X-11452 **(L)** 2-hydroxyacetaminophen sulfate **(M)** X-12696 **(N)** isovalerate **(O)** X-13215 **(P)** X-13671 **(Q)** X-13859.

The MR analysis identified significant associations between plasma metabolites and the risk of PTs. Specifically, 2−hydroxyacetaminophen sulfate (OR=0.97, 95%CI: 0.95-1.00, P=0.024), acetylcarnitine (OR=0.35, 95%CI: 0.19-0.63, P=0.001), isovalerate (OR=0.45, 95%CI: 0.24-0.84, P=0.012), myristate (OR=0.48, 95%CI: 0.27-0.86, P=0.014), pipecolate (OR=0.63, 95%CI: 0.41-0.96, P=0.034), threonate (OR=0.65, 95%CI: 0.43-0.97, P=0.035) showed inverse correlations with PTs’ risk. Conversely, 2−hydroxyisobutyrate (OR=2.27, 95%CI: 1.34-3.84, P=0.002), 3−dehydrocarnitine (OR=2.73, 95%CI: 1.55-4.83, P=0.001), 3−indoxyl sulfate (OR=1.62, 95%CI: 1.03-2.53, P=0.036), citrate (OR=2.17, 95%CI: 1.03-4.55, P=0.040), and glycerate (OR=2.01, 95%CI: 1.01-4.02, P=0.048) were positively related to the risk of PTs. Additionally, six unknown metabolites were discovered to be causally linked to the occurrence of PTs. (**Figures 2**, **3**) Scatter plots illustrating these causal relationships are provided in **Figure 4**. However, reverse MR analysis revealed no causal association between PTs and these plasma metabolites(P<0.05).

### 3.2 MR analysis of 338 CSF metabolites’ impact on PTs

**Figure 5:**
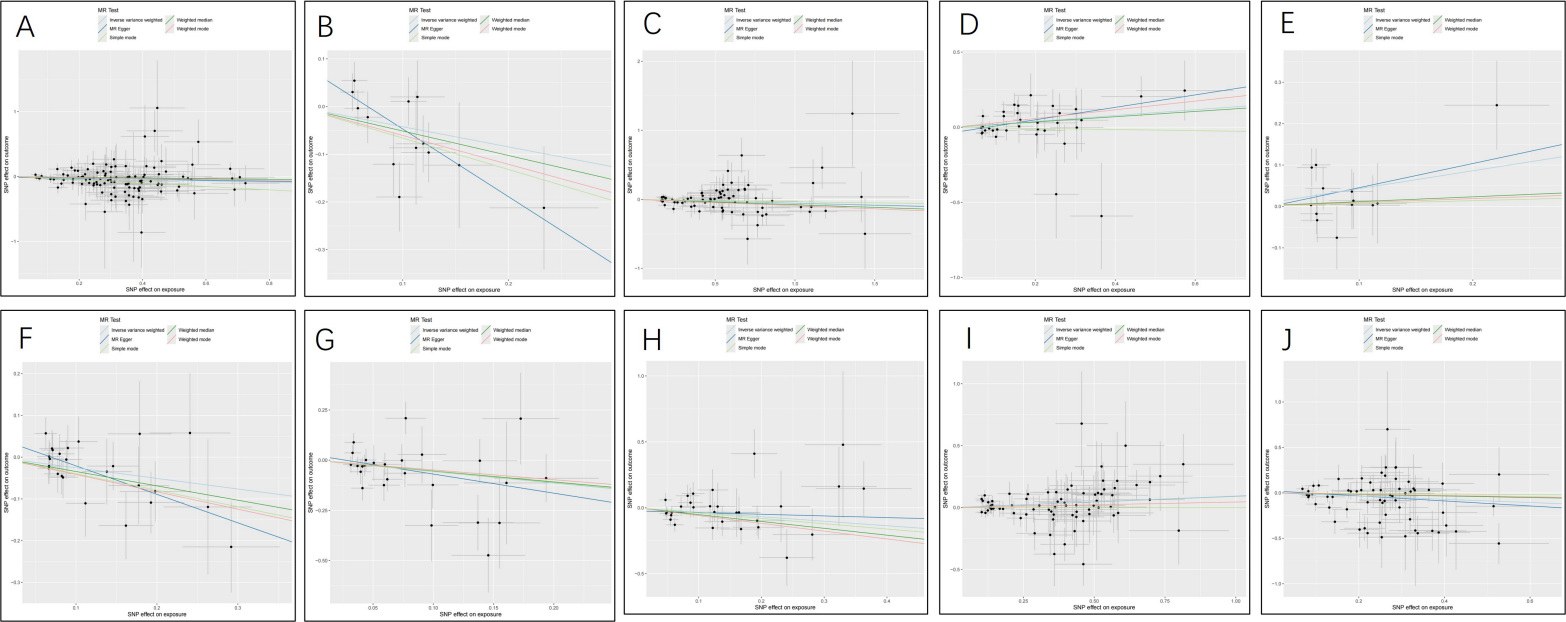
Scatter plots for the causal association between cerebrospinal fluid metabolites and PTs. **(A)** Acetylcarnitine(c2) **(B)** Arabonate/xylonate **(C)** Dimethyl sulfone **(D)** N-acetylglutamate **(E)** N-acetylisoleucine **(F)** N-formylmethionine **(G)** Tryptophan **(H)** Urea **(I)** X-24686 **(J)** X-25109.

The MR analysis displayed significant associations between CSF metabolites and the risk of PTs. Specifically, Acetylcarnitine (c2) (OR=0.90, 95%CI: 0.83-0.97, P=0.005), Arabonate/xylonate (OR=0.65, 95%CI: 0.44-0.98, P=0.037), Dimethyl sulfone (OR=0.95, 95%CI: 0.90-1.00, P=0.034), N-formylmethionine (OR=0.78, 95%CI: 0.63-0.95, P=0.016), Tryptophan (OR=0.57, 95%CI: 0.33-0.96, P=0.036), Urea (OR=0.71, 95%CI: 0.52-0.97, P=0.0353) showed inverse correlations with PTs’ risk. Conversely, N-acetylglutamate (OR=1.21, 95%CI: 1.02-1.44, P=0.027) and N-acetylisoleucine (OR=1.54, 95%CI: 1.04-2.28, P=0.032) were positively associated with the risk of PTs. Additionally, two unknown CSF metabolites were discovered to be causally linked to the occurrence of PTs. (**Table 1**) Scatter plots illustrating these causal relationships are provided in **Figure 5**. However, reverse MR analysis revealed no causal association between PTs and these CSF metabolites (P<0.05).

**Table 1:** The significant IVW results of cerebrospinal fluid metabolites and pituitary tumors.

Exposure	Method	nSNP	b	se	OR (95%CI)	pval
Acetylcarnitine (c2)	IVW	111	-0.11	0.04	0.90(0.83,0.97)	0.005
Arabonate/xylonate	IVW	13	-0.42	0.20	0.65(0.44,0.98)	0.037
Dimethyl sulfone	IVW	76	-0.05	0.03	0.95(0.90,1.00)	0.034
N-acetylglutamate	IVW	34	0.19	0.09	1.21(1.02,1.44)	0.027
N-acetylisoleucine	IVW	13	0.43	0.20	1.54(1.04,2.28)	0.032
N-formylmethionine	IVW	25	-0.25	0.11	0.78(0.63,0.95)	0.016
Tryptophan	IVW	26	-0.57	0.27	0.57(0.33,0.96)	0.036
Urea	IVW	30	-0.34	0.16	0.71(0.52,0.97)	0.032
X-24686	IVW	74	0.09	0.04	1.10(1.02,1.18)	0.016
X-25109	IVW	64	-0.18	0.08	0.84(0.72,0.98)	0.027

### 3.3 Sensitivity analyses

To ensure the robustness of our findings, sensitivity analyses were conducted, confirming the integrity of IVs and hypothesis of the MR pattern through MR-Egger tests and Cochran’s Q. Regarding plasma metabolites and PTs, no evidence of heterogeneity was observed across the seventeen types of plasma metabolites. Except for 2-hydroxyisobutyrate, myristate (14:0) and X-13859, there was no pleiotropy in the other fourteen plasma metabolites. Concerning CSF metabolites and PTs, except for tryptophan and X-25109, there was no heterogeneity in the other eleven CSF metabolites. Except for arabonate/xylonate, there was no pleiotropy in the other eleven CSF metabolites. Furthermore, both leave-one-out and MR-PRESSO analyses detected no underlying instrumental outliers, providing further support for the robustness of our results. Detailed summaries of sensitivity analyses are available in **Tables 2**, **3**, while illustrations of leave-one-out analysis and funnel plots can be discovered in **Supplementary Figures 1**, **2**.

**Table 2:** The sensitivity analysis results of serum metabolites and pituitary tumors.

Exposures	Methods	Q	Q_pval	Ple_pval
2-hydroxyacetaminophen sulfate	IVW	185.18	0.341	0.994
2-hydroxyisobutyrate	IVW	89.21	0.832	0.038
3-dehydrocarnitine	IVW	95.49	0.907	0.134
3-indoxyl sulfate	IVW	55.15	0.803	0.355
acetylcarnitine	IVW	110.88	0.432	0.548
citrate	IVW	180.14	0.156	0.412
glycerate	IVW	107.95	0.154	0.754
isovalerate	IVW	64.58	0.660	0.273
myristate (14:0)	IVW	131.56	0.112	0.021
pipecolate	IVW	90.35	0.224	0.226
threonate	IVW	72.26	0.309	0.278
X-11247	IVW	59.50	0.833	0.527
X-11452	IVW	64.62	0.420	0.914
X-12696	IVW	131.62	0.325	0.418
X-13215	IVW	171.82	0.533	0.300
X-13671	IVW	111.33	0.553	0.579
X-13859	IVW	191.78	0.132	0.026

**Table 3:** The sensitivity analysis results of cerebrospinal fluid metabolites and pituitary tumors.

Exposure	Method	Q	Q_pval	Ple_ pval	Presso_pval
Acetylcarnitine (c2)	MR Egger	90.26	0.904		
	IVW	90.67	0.910	0.526	0.902
Arabonate/xylonate	MR Egger	10.28	0.506		
	IVW	15.67	0.207	0.040	0.260
Dimethyl sulfone	MR Egger	71.87	0.549		
	IVW	71.88	0.581	0.914	0.613
N-acetylglutamate	MR Egger	29.26	0.606		
	IVW	31.63	0.535	0.133	0.574
N-acetylisoleucine	MR Egger	13.40	0.268		
	IVW	13.48	0.335	0.798	0.365
N-formylmethionine	MR Egger	12.75	0.957		
	IVW	16.34	0.876	0.071	0.878
Tryptophan	MR Egger	36.97	0.044		
	IVW	37.91	0.047	0.443	0.059
Urea	MR Egger	32.50	0.255		
	IVW	33.30	0.266	0.413	0.274
X-24686	MR Egger	65.42	0.695		
	IVW	65.42	0.724	0.986	0.746
X-25109	MR Egger	89.89	0.012		
	IVW	90.80	0.012	0.433	0.013

### 3.4 Metabolic pathway analyses

In the metabolic pathway analysis, two significant metabolic pathways primarily involved in PTs were identified. The results showed that the “Arginine biosynthesis” pathway (P = 0.002) and the “Glyoxylate and dicarboxylate metabolism” pathway (P = 0.008) may be involved in the development and occurrence of PTs.

## 4 Discussion

We used serum metabolites, CSF metabolites, and PTs for MR analysis to investigate the causality between metabolites and PTs. In our research, we identified 17 plasma metabolites and 10 CSF metabolites associated with PTs. Among them, 11 metabolites showed significant positive causality with PTs, while 16 metabolites showed significant negative causality with PTs. Our study identified a variety of serum metabolites that may function as biomarkers for PTs. Among them, serum 3-dehydrocarnitine and acetylcarnitine are the most noteworthy.

MR is analogous to randomized controlled trials in nature and uses genetic variations as IVs to make causal inferences. It is able to avoid to some extent the problems of confounders (e.g., overall patient frailty) (18) and reverse causation in traditional observational studies because genes are determined at birth and are not affected by acquired environment or disease. With the increasing application of metabolomics in various diseases within neuroendocrinology and neurosurgery, it has provided new insights and ideas for exploring therapeutic targets and biomarkers for various brain tumors. While metabolomics research on PTs remains in its infancy, numerous researches have consistently demonstrated that metabolomics plays a crucial role and holds promising prospects for studying the mechanisms of PTs (5, 19).

Metabolites play a crucial role in living organisms, participating in processes such as energy metabolism, cell signaling, and maintaining intracellular stability. Abnormal changes in metabolites can reflect the development of specific diseases. Metabolomics has shown that different subtypes of pituitary adenomas exhibit distinct metabolic profiles. Previous studies have identified differences in the levels of metabolites such as deoxycholic acid, pyridoxine, and methyl 3-adipate in ACTH adenomas (20). In prolactinomas, downregulation of N-acetylaspartate, phosphoethanolamine, and inositol expression, along with upregulation of glutamine, aspartate, and glutamate expression, has been observed (21). In invasive non-functioning pituitary adenomas, the levels of creatinine, desipramine, taurine, hypotaurine, lactate, and succinic acid were upregulated, whereas the levels of cis-11-eicosatetraenoic acid, glyceric acid, docosahexaenoic acid, arachidonic acid, hypoxanthine, lysine, linoleic acid, xanthine, valine, uracil, and oleic acid were downregulated (8). In contrast, differences in amino acids, lipids and glucose metabolites were also present in acromegaly (19). Our discovery that acetylcarnitine is inversely associated with PTs suggests that it could represent a significant therapeutic target for PTs. Acetylcarnitine, a derivative of carnitine, plays a crucial role in multiple neural pathways and has demonstrated utility in treating various neurological disorders, including dementia, cerebral ischemia, and neuroblastoma (22–25). Acetyl L-carnitine has been proposed to modulate the tumor microenvironment through its impact on the target glandular axis and subsequent neuroendocrine secretion (26). 3-Dehydrocarnitine, belonging to the carnitine family, functions as an intermediate in the degradation of carnitine. Carnitine has traditionally been linked with fatty acid metabolism. Metabolites of fatty acids might influence PTs growth by modulating the release of inflammatory factors and neurotransmitters from pertinent signaling pathways (6, 27).

Lipid metabolism is pivotal in the initiation and advancement of PTs (28). Lipid metabolism represents a significant component of the metabolic reprogramming observed in tumor cells (29). Large quantities of fatty acids can either be utilized for constructing cell membranes or oxidized to generate energy (30, 31). In addition, lipids serve not only as structural components and sources of energy for tumor cells but also regulate cell growth, differentiation, and proliferation through signaling pathways (32). Our study indicates a causal association between acetylcarnitine, myristate, isovalerate, 2-hydroxyisobutyric acid, and 3-dehydrocarnitine—lipids and their related compounds—and the development of pituitary tumors. Earlier research has also linked taurine and glyceric acid to IGF-1 levels, suggesting their potential as metabolomic biomarkers for active acromegaly (33). Additionally, integrated protein-metabolite pathway analyses demonstrated significant enrichment of multiple metabolites within the fatty acid metabolic pathway in ACTH-secreting pituitary adenomas (34). Thus, these results collectively indicate that lipid metabolism could indeed play a significant role in the development of pituitary tumors, potentially influencing metabolic reprogramming and signaling pathways like the phosphatidylinositol-3-kinase (PI3K)/Akt pathway (35–38). Furthermore, fluctuations in the levels of these lipid metabolites in pituitary tumors can serve as biomarkers for tumor diagnosis and treatment.

The relationship between amino acid metabolism and PTs is complex (39). Our study suggests that some amino acid metabolites such as Arabonate, N-formylmethionine, Tryptophan, N-acetylglutamate and N-acetylisoleucine are causally associated with the development of PTs. Notably, N-acetylglutamate is related to the glutamine metabolic pathway. One of the most important mechanisms linking amino acid metabolism and PTs is glutamine metabolism, which is altered in pituitary tumorigenesis and may vary across different clinical types of PTs (40). Glutamine provides ATP to tumor cells, supplies critical precursors for nucleotides, and helps tumor cells survive in hostile environments through the synthesis of glutathione (41). Additionally, glutamine metabolism is intricately linked to the mTOR signaling pathway, which is crucial for tumor cell proliferation and growth (42, 43). Furthermore, certain amino acids, such as tryptophan, may influence the pituitary gland via the neuroendocrine pathway, indirectly affecting pituitary cell proliferation and tumor formation (44).

Further transcriptome analysis suggests that ligands associated with 3-dehydrocarnitine, including PPARα, AMPK, SREBP, ChREBP, PGC-1α, and SIRT, may be involved in the signaling pathways of gonadotroph tumors, while PPARα, AMPK, SREBP, and ChREBP ligands may play a role in the signaling pathways of prolactinoma. Similarly, ligands associated with acetylcarnitine, such as PPARα, PGC-1α, SIRT1, PPARδ, SREBP-1c, LXRα/β, and PPARγ, are likely involved in the signaling of gonadotroph tumors. Additionally, PPARα, PGC-1α, and FOXO1 ligands linked to acetylcarnitine may participate in the signaling pathways of prolactinoma, while SREBP-1c, LXRα/β, PPARγ, HIF-1α, PPARα, PGC-1α, and FOXO1 are likely involved in the signaling of nonfunctional pituitary tumors (45). These transcriptome ligands jointly regulate the expression of related metabolites and genes, modulating signaling pathways that ultimately impact tumor development and progression (**Supplementary Table 1**).

The arginine biosynthetic pathway consists of a series of chemical reactions that convert amino acids into arginine. Arginine is an essential amino acid with several cellular functions, including protein synthesis and cell proliferation (46). Tumor cells may upregulate the arginine synthesis pathway to meet the demands of rapid proliferation, particularly in pituitary tumors (47). Furthermore, arginine metabolism is closely related to immune regulation (48). Pituitary tumor cells can inhibit T-cell activity by depleting arginine, thereby evading the immune system’s attack (49). Key enzymes in the arginine synthesis pathway, such as arginase and nitric oxide synthase, are upregulated in pituitary tumors and may serve as diagnostic markers or therapeutic targets. Glyoxylate is metabolized via the glyoxylate cycle or glycine pathway to produce the intermediate product glyoxylate, which can be further metabolized to oxalate or converted into other intermediates that enter the tricarboxylic acid cycle. The accumulation of glyoxylate and its metabolites may influence the oxidative stress levels and metabolic state of pituitary tumor cells, thereby promoting tumor growth and survival (50). Dicarboxylic acids, including oxalic acid, fumaric acid, and succinic acid, are crucial intermediates of the TCA cycle, involved in energy production and cellular metabolism (51). Pituitary tumor cells may undergo metabolic reprogramming in the dicarboxylate metabolic pathway, promoting cell proliferation and anti-apoptosis by affecting ATP production, redox state, and the accumulation of metabolic by-products (38). Investigating abnormalities in the arginine metabolic pathway, as well as the glyoxylate and dicarboxylate metabolic pathways, may help identify pituitary tumor-specific biomarkers for diagnosis and monitoring disease progression. Drugs targeting these metabolic pathways could be utilized in the treatment of pituitary tumors. For example, inhibiting key enzymes in these pathways may reduce the energy supply and growth advantage of tumor cells. Additionally, by modulating oxidative stress levels, the proliferation of tumor cells may be inhibited, and apoptosis may be promoted.

Our study examined the causality of serum and CSF metabolites with PTs, identifying numerous metabolites that could serve as potential biomarkers for PTs. Notably, serum 3-dehydrocarnitine and acetylcarnitine emerged as the most promising biomarkers. However, several limitations must be acknowledged. Firstly, our MR analyses were based on metabolite GWAS data with a limited sample size, necessitating the collection of more samples and metabolite species across diverse populations to accurately elucidate the relationship between metabolites and PTs. Secondly, our study represents a preliminary exploration; thus, further experimental researches are desired to validate these results and investigate underlying mechanisms in greater detail. Thirdly, our findings may be specific to European populations and might not be generalizable to other ethnic groups. Lastly, due to the exploratory nature of our study, multiple testing adjustments were not applied; however, we ensured the robustness of our results by employing multiple MR algorithms.

## 5 Conclusions

In conclusion, our comprehensive analysis of 486 plasma metabolites and 338 CSF metabolites successfully identified 27 metabolites associated with PTs. These findings underscore the potential of plasma and CSF metabolites, particularly serum 3-dehydrocarnitine and acetylcarnitine, as biomarkers for PTs. This research opens promising avenues for early detection and risk assessment of PTs.

## Data Availability

The original contributions presented in the study are included in the article/**Supplementary Material**. Further inquiries can be directed to the corresponding author.
