# Supplementary material for: Elucidating the causal links between plasma and cerebrospinal fluid metabolites and pituitary tumors: a Mendelian randomization analysis
Source: Front Endocrinol (Lausanne). 2024 Nov 28;15:1460278. doi: 10.3389/fendo.2024.1460278 (PMC11634583; doi:10.3389/fendo.2024.1460278)
Supplement: Supplementary file 1 [file DataSheet1.docx]

Supplement Figure 1:

1. Funnel plots for the causal association between plasma metabolites and PTs.


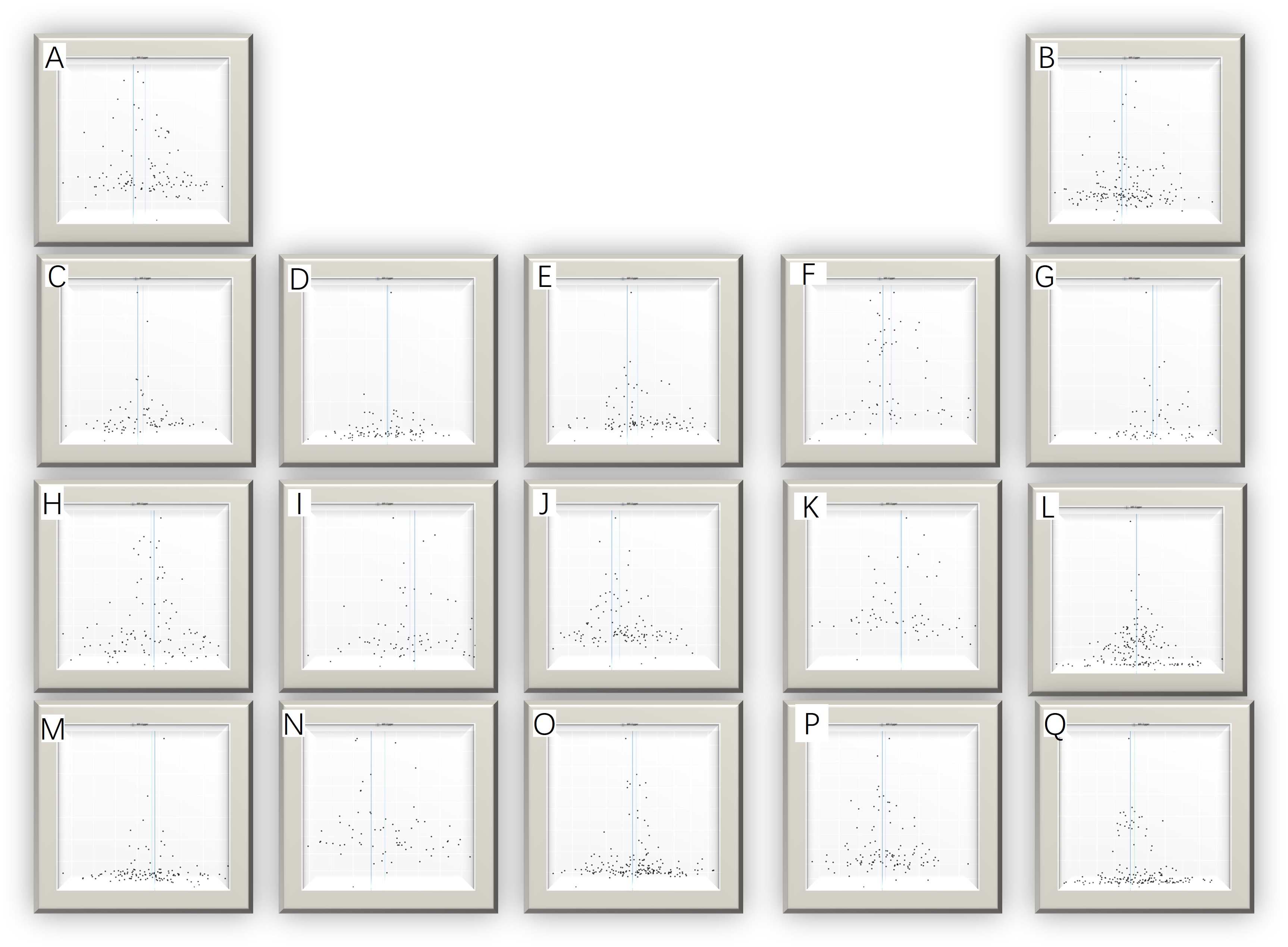


1. Leave-one-out plots for the causal association between cerebrospinal fluid metabolites and PTs.


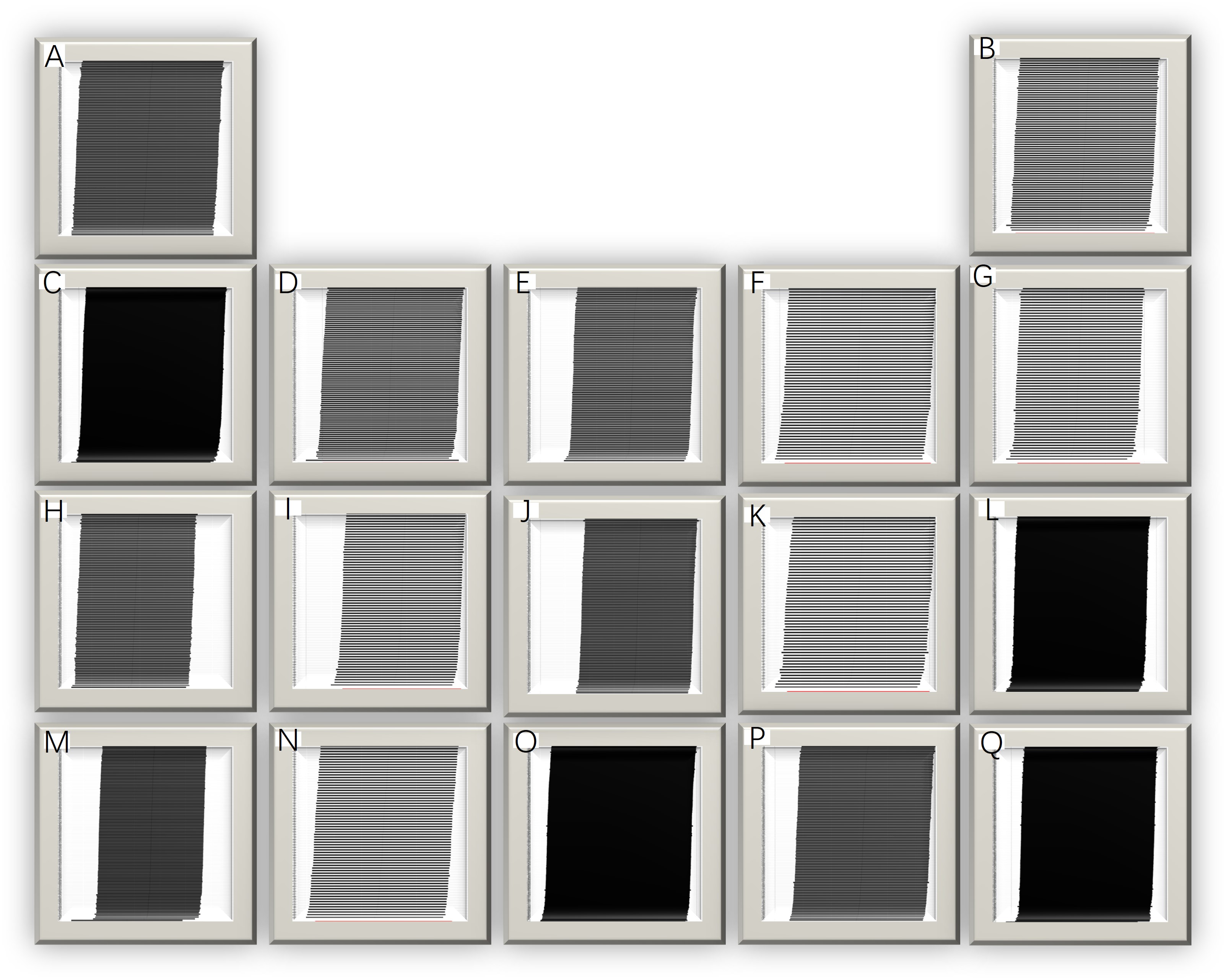


A myristate (14:0) B pipecolate C citrate D glycerate E 2-hydroxyisobutyrate F 3-indoxyl sulfate G threonate H acetylcarnitine I X-11247 J 3-dehydrocarnitine K X-11452 L 2-hydroxyacetaminophen sulfate M X-12696 N isovalerate O X-13215 P X-13671 Q X-13859
