# Supplementary material for: Elucidating the causal links between plasma and cerebrospinal fluid metabolites and pituitary tumors: a Mendelian randomization analysis
Source: Front Endocrinol (Lausanne). 2024 Nov 28;15:1460278. doi: 10.3389/fendo.2024.1460278 (PMC11634583; doi:10.3389/fendo.2024.1460278)
Supplement: Supplementary file 2 [file DataSheet2.docx]

Supplement Figure 2:

1. Funnel plots for the causal association between cerebrospinal fluid metabolites and PTs.


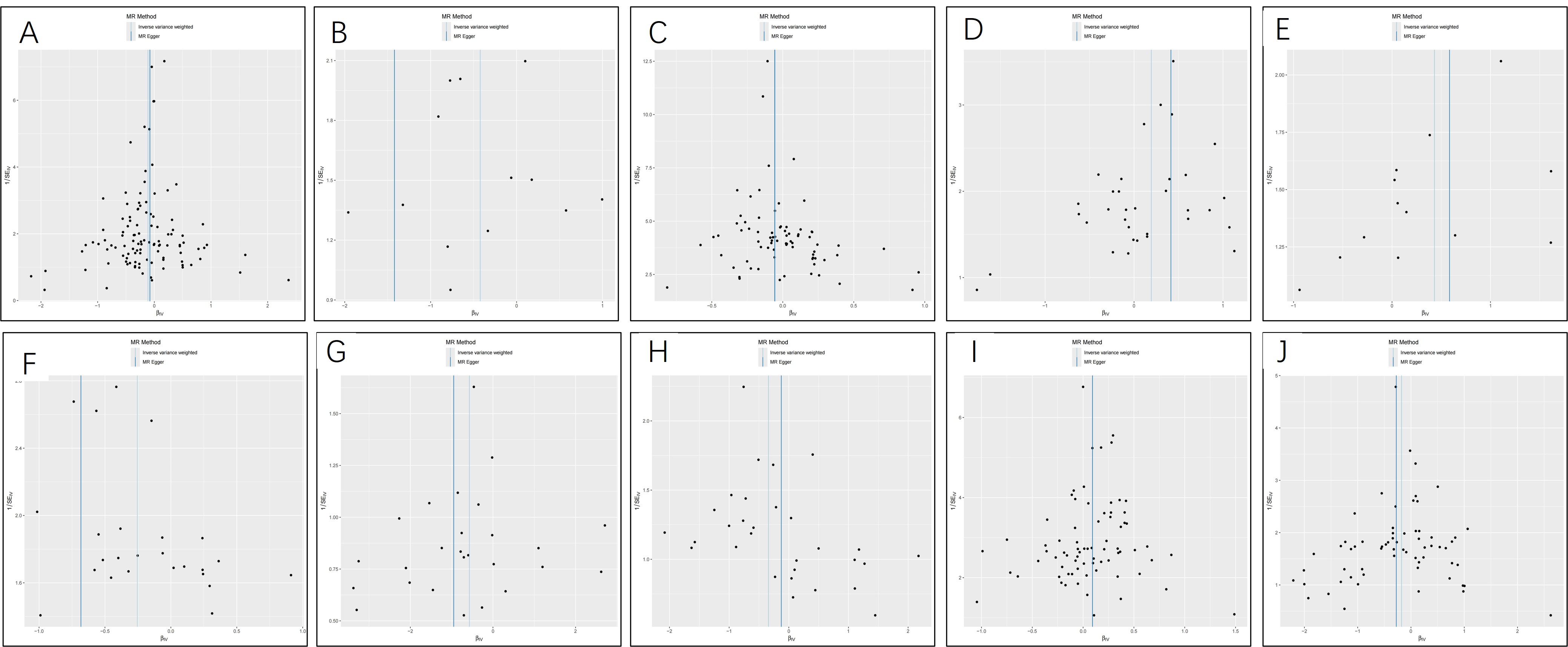


1. Leave-one-out plots for the causal association between cerebrospinal fluid metabolites and PTs.


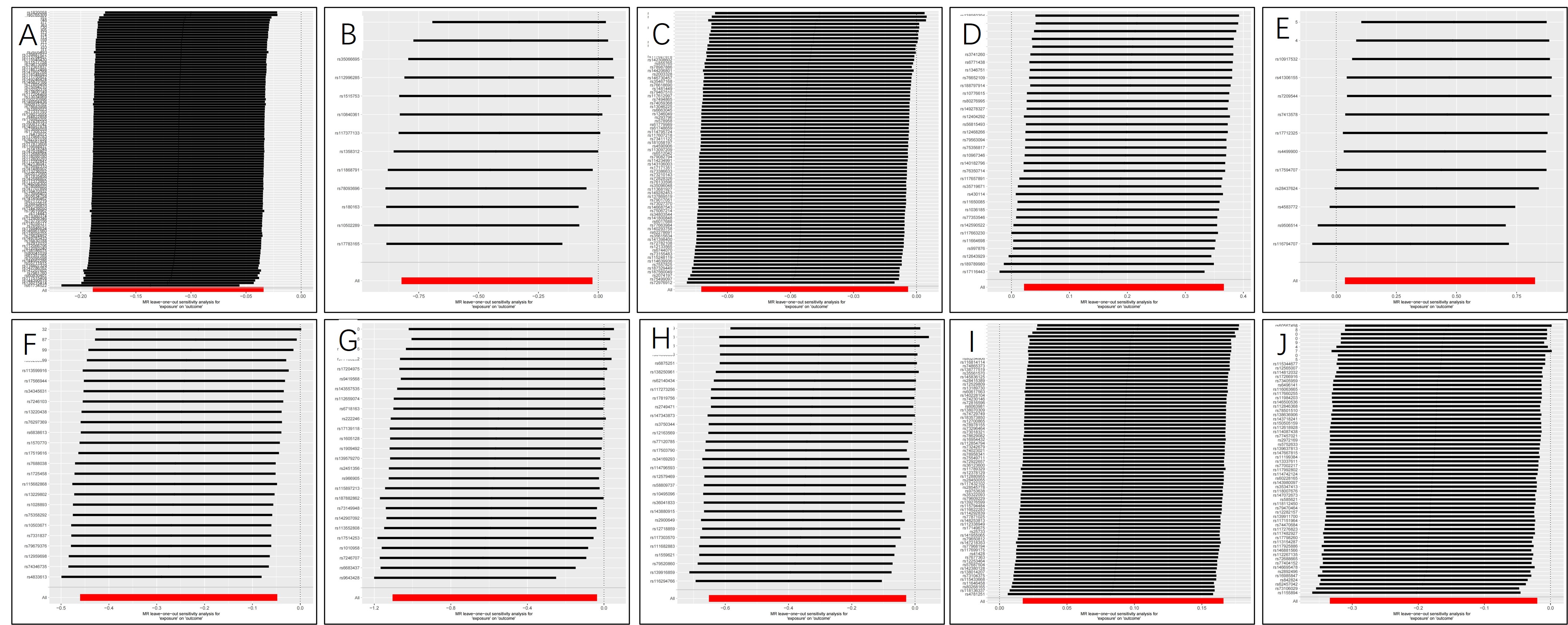


1. Forest plots for the causal association between cerebrospinal fluid metabolites and PTs.


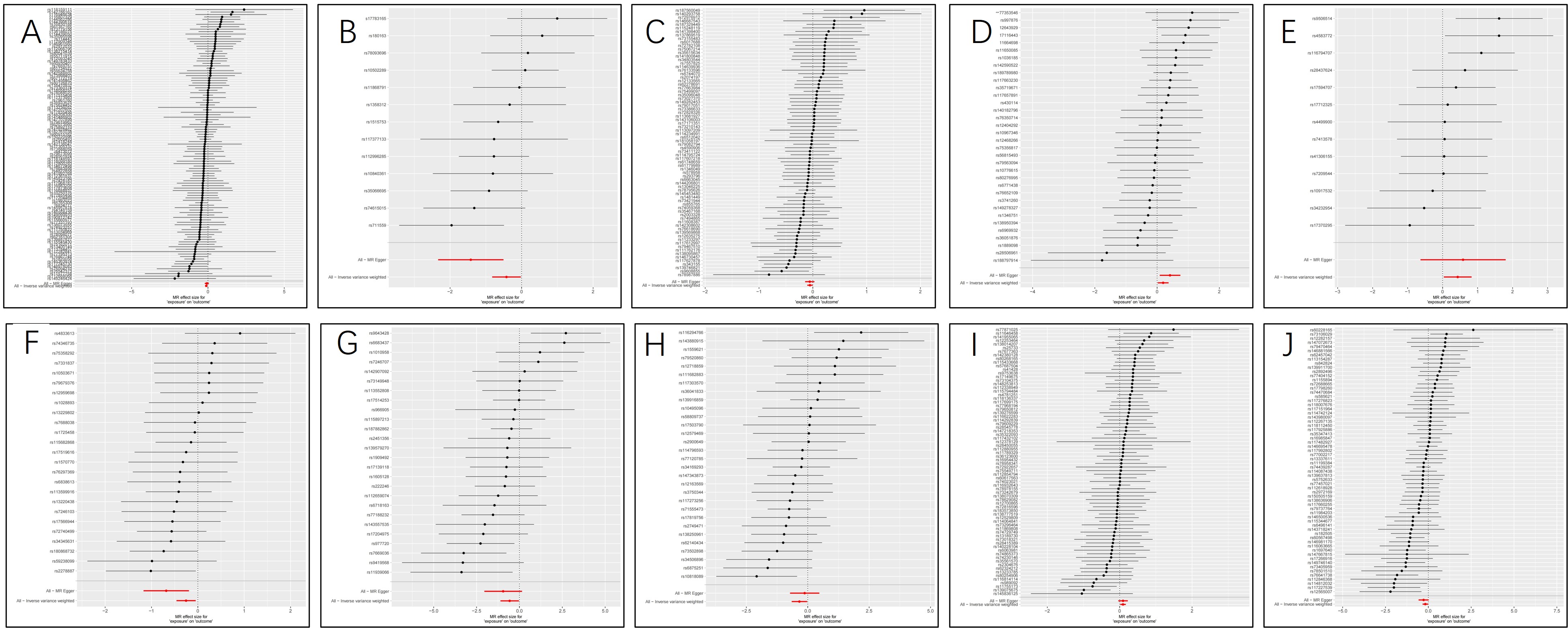


A Acetylcarnitine(c2) B Arabonate/xylonate C Dimethyl sulfone D N-acetylglutamate E N-acetylisoleucine F N-formylmethionine G Tryptophan H Urea I X-24686 J X-25109
