# Supplementary material for: Elucidating the causal links between plasma and cerebrospinal fluid metabolites and pituitary tumors: a Mendelian randomization analysis
Source: Front Endocrinol (Lausanne). 2024 Nov 28;15:1460278. doi: 10.3389/fendo.2024.1460278 (PMC11634583; doi:10.3389/fendo.2024.1460278)
Supplement: Supplementary file 3 [file Table1.docx]

**Supplement Table 1: A summary table of metabolites, genes, transcriptome ligands, and their roles in different types of pituitary tumors.**

| **Metabolites** | **Gene** | **Transcriptome ligand** | **Types of PTs** |
| --- | --- | --- | --- |
| **3-dehydrocarnitine** | CPT1A | PPARα, AMPK, SREBP, ChREBP | Gonadotroph tumors，prolactinoma |
|  | CPT2 | PPARα，PGC-1α，SREBP-1c |  |
|  | CRAT | PPARα, PGC-1α, SIRT1 | Gonadotroph tumors |
| **Acetylcarnitine (c2)** | CRAT | PPARα, PGC-1α, SIRT1 | Gonadotroph tumors |
|  | CPT1A | PPARα、PGC-1α、FOXO1 | Gonadotroph tumors, prolactinoma |
|  | CPT1B | PPARδ、PPARα、PGC-1α和FOXO1 |  |
|  | SLC22A5 | PPARα、PPARδ、PGC-1α、FOXO1 | Gonadotroph tumors |
|  | ACAT1 | SREBP-1c、LXRα/β、PPARγ | Gonadotroph tumors; Nonfunctional pituitary tumors |
|  | PDHA1 | HIF-1α、PPARα、PGC-1α和FOXO1 | Nonfunctional pituitary tumors |
